# Lysine and Leucine Deficiencies Affect Myocytes Development and IGF Signaling in Gilthead Sea Bream (*Sparus aurata*)

**DOI:** 10.1371/journal.pone.0147618

**Published:** 2016-01-25

**Authors:** Sheida Azizi, Mohammad Ali Nematollahi, Bagher Mojazi Amiri, Emilio J. Vélez, Esmail Lutfi, Isabel Navarro, Encarnación Capilla, Joaquim Gutiérrez

**Affiliations:** 1 Department of Fisheries Sciences, Faculty of Natural Resources, University of Tehran, Karaj, Iran; 2 Departament de Fisiologia i Immunologia, Facultat de Biologia, Universitat de Barcelona, 08028, Barcelona, Spain; Institut National de la Recherche Agronomique (INRA), FRANCE

## Abstract

Optimizing aquaculture production requires better knowledge of growth regulation and improvement in diet formulation. A great effort has been made to replace fish meal for plant protein sources in aquafeeds, making necessary the supplementation of such diets with crystalline amino acids (AA) to cover the nutritional requirements of each species. Lysine and Leucine are limiting essential AA in fish, and it has been demonstrated that supplementation with them improves growth in different species. However, the specific effects of AA deficiencies in myogenesis are completely unknown and have only been studied at the level of hepatic metabolism. It is well-known that the TOR pathway integrates the nutritional and hormonal signals to regulate protein synthesis and cell proliferation, to finally control muscle growth, a process also coordinated by the expression of myogenic regulatory factors (MRFs). This study aimed to provide new information on the impact of Lysine and Leucine deficiencies in gilthead sea bream cultured myocytes examining their development and the response of insulin-like growth factors (IGFs), MRFs, as well as key molecules involved in muscle growth regulation like TOR. Leucine deficiency did not cause significant differences in most of the molecules analyzed, whereas Lysine deficiency appeared crucial in IGFs regulation, decreasing significantly IGF-I, IGF-II and IGF-IRb mRNA levels. This treatment also down-regulated the gene expression of different MRFs, including Myf5, Myogenin and MyoD2. These changes were also corroborated by a significant decrease in proliferation and differentiation markers in the Lysine-deficient treatment. Moreover, both Lysine and Leucine limitation induced a significant down-regulation in FOXO3 gene expression, which deserves further investigation. We believe that these results will be relevant for the production of a species as appreciated for human consumption as it is gilthead sea bream and demonstrates the importance of an adequate level of Lysine in fishmeal diet formulation for optimum growth.

## Introduction

Gilthead sea bream (*Sparus aurata* L.*)* is a subtropical species very important for aquaculture production, being in the Mediterranean one of the main species cultured. However, optimization of its production requires a better knowledge of growth regulation and improvement in diet formulation, looking for best performance and the sustainability of aquaculture. For this purpose, in the last years, scientists have made great efforts to replace fish meal for plant protein formulations in the aquafeeds for this species [1–3]. One of the key points to take into account on these replacements is the different amino acids (AA) profile between plant proteins and fish meal. These profile differences may cause changes in absorption, modifying the AA plasma levels and increasing the endogenous protein mobilization through proteolysis or altering the hepatic metabolism [4]. These problems can be solved by supplementing feeds with crystalline AA to cover the nutritional requirements for each species [5]. In a study in rainbow trout, Snyder et al. [6] found that feeding a diet based in plant protein and supplemented with crystalline AA, differentially induced changes in muscle gene expression, suggesting a myogenic reduced potential due to plant protein AA profile. In this sense, Lysine and Methionine seem to be among the most important limiting essential AA in fish, as it has been demonstrated that supplementation with Lysine improves growth in rainbow trout [7, 8], and other species such as yellow perch [9]. On the contrary, diets with imbalanced Lysine to Arginine ratios reduced growth performance in juvenile cobia [10]. The case of Methionine has been well studied, and supplementation with this AA also improves growth in rainbow trout [11, 12]. Furthermore another essential AA, Leucine and its catabolites participate in disease protection [13] but an excess of Leucine can also have toxic effects affecting body composition and growth in rainbow trout [14].

In addition to nutritional signals, hormones are also key regulators of muscle growth, where the growth hormone (GH)—insulin-like growth factors (IGFs) axis plays a major role [15–18]. The IGFs act as systemic and paracrine/autocrine factors to promote tissue growth [19–22]. These effects could be modulated by controlling availability and activity of IGFs through different IGF binding proteins (IGFBPs 1–6), by means of the IGF-I receptors (IGF-IRs) [23, 24], or via several transduction pathways [25–28]. The target of rapamycin (TOR) is the link between the hormonal signaling (IGFs) and AA, which are important nutrients stimulating protein synthesis by activating TOR [29, 30]. In mammals it is well-known that Leucine supplementation stimulates protein synthesis trough TOR in skeletal muscle [31], while Leucine starvation provokes changes in gene expression including inhibition of TOR [32]. In rainbow trout, also TOR gene expression is increased in parallel to different levels of dietary Methionine, as well as the GH-IGFs axis is regulated [12]. In contrast, Wacyk et al. [4] found no differences in TOR expression in rainbow trout muscle in an experiment of fish meal replacement for plant protein, whereas the expression of red-1, a gene known to repress TOR function, was increased in the fish fed the plant based diet.

Using *in vitro* models, Averous et al. [33] investigated particularly how Leucine limitation regulates myogenic factors expression in mice primary satellite cells. In teleosts, similar studies using a cocktail of AA have highlighted an essential role for them in the cellular events required during myocyte development [34–39]. Although so far, most studies in fish on specific AA requirements have focused on their effects at hepatic levels, as is the case of Lysine and Leucine in rainbow trout hepatocytes [40].

A gilthead sea bream *in vitro* model of myocyte cells has been developed by our group as a way to investigate specific physiological conditions affecting myogenesis and at the same time saving on sacrifices of bigger fish and avoiding the stressful conditions that an experimental *in vivo* treatment may cause [27]. Furthermore, this model has the advantage of analyzing just the specific effect of the AA studied, without influences of appetite changes, reduction on feed intake and subsequently, weight gain and growth rate, as it has been related in some experiments [4, 6, 41, 42]. Studies by our group [28, 43, 44] showed the metabolic effects of IGFs on AA metabolism, correlation between IGFs system with AA signal and eventually stimulation of growth. Recently, Vélez et al. [39] have demonstrated the effects of AA supplementation on the TOR signaling pathway at both gene and protein levels in myocytes, and recently Azizi et al. [45] have characterized the two IGF-I receptors in gilthead sea bream, and also analyzed the effects of IGFs stimulation on the GH-IGFs axis-related genes, myogenic regulatory factors (MRFs) expression and TOR pathway. However, the effects of AA deficiencies in these molecules are completely unknown.

Thus, this study aimed to provide new information on the specific effects of Lysine and Leucine deficiencies in gilthead sea bream myocytes cultured *in vitro*, examining through their development, the response of IGF system and MRFs genes, as well as the expression of key molecules involved on muscle function like TOR. We believe that these results will be relevant in a species as appreciated for human consumption and aquaculture as it is gilthead sea bream.

## Material and Methods

### 2.1. Experimental animals and ethical statement

Juvenile gilthead sea bream (*Sparus aurata* L.) obtained from a commercial hatchery in the north of Spain were maintained in the facilities of the Faculty of Biology at the University of Barcelona with a sea water recirculation system at a temperature of 21±1°C under 12:12 h light cycle. Fish were fed *ad libitum* twice a day with a commercial diet (Skretting, Burgos, Spain). Food was held for 24 h before sampling. All procedures were approved by the Ethics and Animal Care Committee of the University of Barcelona following the European Union, Spanish and Catalan Governments-assigned principles and legislations (permit numbers CEEA 168/14 and DAAM 7749).

### 2.2. Myocyte primary cell culture and treatments

Juveniles of gilthead sea bream with body weight ranging from 5 to 15 g were sacrificed by a blow to the head and satellite cells were isolated as described previously [27]. Isolated cells from 4 independent cultures were plated at a density of 1.5–2·10^5^ cells/cm^2^ into 6-well plates for gene expression analyses, and in 12-well plates with or without coverslips for the proliferation or immunocytochemistry assays, respectively. Then, cells were cultured in Dulbecco’s Modified Eagle Medium (DMEM) supplemented with 0.11% NaCl, 10% fetal bovine serum (FBS) and 1% antibiotic/antimycotic solution at 23°C. All tissue culture reagents, unless noted otherwise were purchased from Sigma-Aldrich (Tres Cantos, Spain) and all plastic ware was from Nunc (Labclinics, Barcelona, Spain).

For the Lysine and Leucine deficiency experiments we prepared 3 different media: control, without Lysine (Lys) and without Leucine (Leu). All were made from DMEM/F12HAM (3.15 g/l glucose) (D9785, Sigma-Aldrich, Tres Cantos, Spain) devoid of Lysine and Leucine as a base media. Then, each experimental medium was prepared adding the corresponding AA, Lysine, Leucine or both according to the manufacturer’s indications plus 10% FBS and 1% antibiotic/antimycotic solution. The final concentration of each AA of interest was measured in both, the FBS and the different media by cation-exchange chromatography followed by post-column derivatization with ninhydrin and UV detection using a Biochrom 30 analyzer at the Scientific and Technological Centers of the University of Barcelona. In the case of the control condition, there was a 398.0 μM concentration of Lysine and a 389.6 μM of Leucine. In the respective experimental media conditions, concentration of Lysine was 24.7 μM, and for Leucine 24.2 μM provided by the 10% FBS, representing a reduction of 93.8% on each case respect to the control condition. For the proliferation experiments, the media was changed one day after plating. Then, the myocytes were maintained on each corresponding media and sampled at days 2, 4 or 8 of culture development for proliferation MTT assay and at days 4 and 8 for proliferating cell nuclear antigen (PCNA) immunocytochemistry. For the gene expression analyses the media was changed at day 1 after plating for the samplings at days 2 and 4 and at day 7 of culture for the sampling at day 8. Cultures development was monitored daily using an Axiovert 40C inverted microscope (Zeiss, Germany) and images were captured with a Canon EOS 1000D digital camera.

### 2.3. Real-time quantitative PCR (qPCR)

To obtain RNA samples cells were recovered from 2 wells per condition with 1 mL TRI reagent solution (Applied Biosystems, Alcobendas, Spain) and the RNA was extracted according to the manufacturer’s protocol. Total RNA quantification and quality assessment were done using a NanoDrop 2000 (Thermo Scientific, Alcobendas, Spain) and running a 1% agarose gel electrophoresis. Then, 500 ng of total RNA were treated with DNase I (Life Technologies, Alcobendas, Spain) and reverse transcribed with the Transcriptor First Strand cDNA synthesis Kit (Roche, Sant Cugat del Valles, Spain) following the manufacturer’s recommendations. Next, qPCR analyses including all the negative controls and preliminary tests (e.g. to determine primer specificity or absence of primer-dimer formation) were performed as described previously [39, 46, 47] using a CFX384^TM^ Real-Time System (Bio-Rad, El Prat de Llobregat, Spain). Primer sequences and specific annealing temperatures are presented in Table 1. Primers for CHOP and AS were designed using Net primer (http://www.premierbiosoft.com/netprimer/) with the nucleotide sequences retrieved from the Nutrigroup-IATS gilthead sea bream nucleotide database at www.nutrigroup-iats.org/seabreamdb [48]. Transcript abundance based on the Pfaffl method was calculated relative to the geometric mean of the reference genes elongation factor 1α (EF1α) and ribosomal protein S18 (RPS18) as they were both stably expressed.

**Table 1:** Primers used in the qPCR analyses.

Gene	Primer sequences (5’-3’)	Ta (°C)	Accession No.
*EF1a*	**F:**CTTCAACGCTCAGGTCATCAT **R:**GCACAGCGAAACGACCAAGGGGA	60	AF184170
*RPL27*	**F:**AAGAGGAACACAACTCACTGCCCCAC **R:**GCTTGCCTTTGCCCAGAACTTTGTAG	68	AY188520
*RPS18*	**F:**GGGTGTTGGCAGACGTTAC **R:**CTTCTGCCTGTTGAGGAACCA	60	AM490061.1
*IGF-I*	**F:**ACAGAATGTAGGGACGGAGCGAATGGAC **R:**TTCGGACCATTGTTAGCCTCCTCTCTG	60	AY996779 EF688015 EF688016
*IGF-II*	**F:**TGGGATCGTAGAGGAGTGTTGT **R:**CTGTAGAGAGGTGGCCGACA	60	AY996778
*IGF-IRa*	**F:**AGCATCAAAGACGAACTGG **R:**CTCCTCGCTGTAGAAGAAGC	55	KT156846
*IGF-IRb*	**F:**GCTAATGCGAATGTGTTGG **R:**CGTCCTTTATGCTGCTGATG	55	KT156847
*IGFBP-4*	**F:**TCCACAAACCAGAGAAGCAA **R:**GGGTATGGGGATTGTGAAGA	60	F5T95CD02JMZ9K
*IGFBP-5*	**F:**TTTCTCTCTCGGTGTGC **R:**TCAAGTATCGGCTCCAG	60	AM963285
*Pax7*	**F:**ATGAACACTGTCGGCAACG **R:**AGGCTGTCCACACTCTTGATG	64	JN034418
*Myf5*	**F:**CTACGAGAGCAGGTGGAGAACT **R:**TGTCTTATCGCCCAAAGTGTC	64	JN034420
*Myogenin*	**F:**CAGAGGCTGCCCAAGGTCGAG **R:**CAGGTGCTGCCCGAACTGGGCTCG	68	EF462191
*MRF4*	**F:**CATCCCACAGCTTTAAAGGCA **R:**GAGGACGCCGAAGATTCACT	60	JN034421
*MyoD1*	**F:**TTTGAGGACCTGGACCC **R:**CTTCTGCGTGGTGATGGA	60	AF478568.1
*MyoD2*	**F:**CACTACAGCGGGGATTCAGAC **R:**CGTTTGCTTCTCCTGGACTC	60	AF478569
*PCNA*	**F:**TGTTTGAGGCACGTCTGGTT **R:**TGGCTAGGTTTCTGTCGC	58	NM_131404.2
*MHC*	**F:**AGCAGATCAAGAGGAACAGCC **R:**GACTCAGAAGCCTGGCGATT	58	AY550963.1
*AKT2*	**F:**GCTCACCCCACTCTTCAGAC **R:**AAATTGGGAAATGTGCTTGC	60	ERA047531
*ERK2*	**F:**AAAGCTCTGGACCTGTTGGA **R:**TCATCCAGCTCCATGTCAAA	60	ERA047531
*TOR*	**F:**CAGACTGACGAGGATGCTGA **R:**AGTTGAGCAGCGGGTCATAG	60	---
*FOXO3*	**F:**CAGCAGCCTGGAGTGTGATA **R:**CCAGCTCTGAGAGGTCTGCT	60	---
*4EBP1*	**F:**CCAACCTGCGACTCATCTCT **R:**GTTCCTCTCATCCTCCCACA	60	---
*70S6K*	**F:**GCACCAGAAAGGCATCATCT **R:**AAGGTGTGGGTCACTGTTCC	60	---
*ATF4*	**F:**TCGCTCGATTTGCCGAAATG **R:**TGGCTGGATGCACTGTTTTG	60	JQ308824.1
*AS*	**F:**ACTGCTGTTTTGGCTTCCAC **R:**ACTTCTTGATGCGCAAAGGC	58	---
*CHOP*	**F:**AAGAAGTCGGTGGACAGGTTC **R:**AGTTGCGCATCTTGGCTTTG	58	---

F: forward; R: reverse; Ta: annealing temperature.

### 2.4. Proliferation assay: MTT

Metabolically active cells reduce yellow methylthiazolyldiphenyl-tetrazolium bromide (MTT) by function of mitochondrial dehydrogenase enzymes producing purple formazan that can be quantified by a spectrophotometer as a reliable way to examine cell proliferation. MTT was added to each well for the last 14 h of treatment before sampling and then, cells were washed, the formazan crystals resuspended and the absorbance reads and calculations performed as previously described using fish cells [49].

### 2.5. Immunocytochemistry

Cell proliferation was analyzed by immunostaining using a commercial PCNA staining kit (Cat. No. 93–1143, Life Technologies, Alcobendas, Spain) as related before [39]. Briefly, cells were washed and fixed at room temperature with 4% paraformaldehyde and postfixed with ethanol. Later, following the suggested manufacturer’s protocol, coverslips were blocked and incubated with anti-PCNA primary antibody and a biotinylated secondary antibody. Finally, cells were dehydrated in a graded alcohol series and mounted with histomount. The amount of PCNA-positive cells was calculated by dividing the PCNA-positive stained cells by the total number of nuclei in 14 images per coverslip containing a total of 400–1300 cells using the ImageJ software (National Institutes of Health, Bethesda, MD, USA). Digital images were acquired with a CC2 camera coupled to a microscope at 40X using analySIS (Soft Imaging System) software. All images were analyzed by the same researcher.

### 2.6. Statistical analysis

All statistical analyses were performed using the package IBM SPSS statistics v.20 (IBM, Chicago, IL, USA). Data was tested for normality using the Shapiro-Wilk test and for homogeneity of variances by Levene’s test. Differences through time within treatments were analyzed by a One-way ANOVA. Differences between experimental treatments at each time respect to the control condition were assessed by a Student’s t-test. When normality was not observed the non-parametric tests Kruskal-Wallis followed by Mann-Whitney U test were applied. In all cases results were considered statistically significant at p<0.05.

## Results

**Fig 1:**
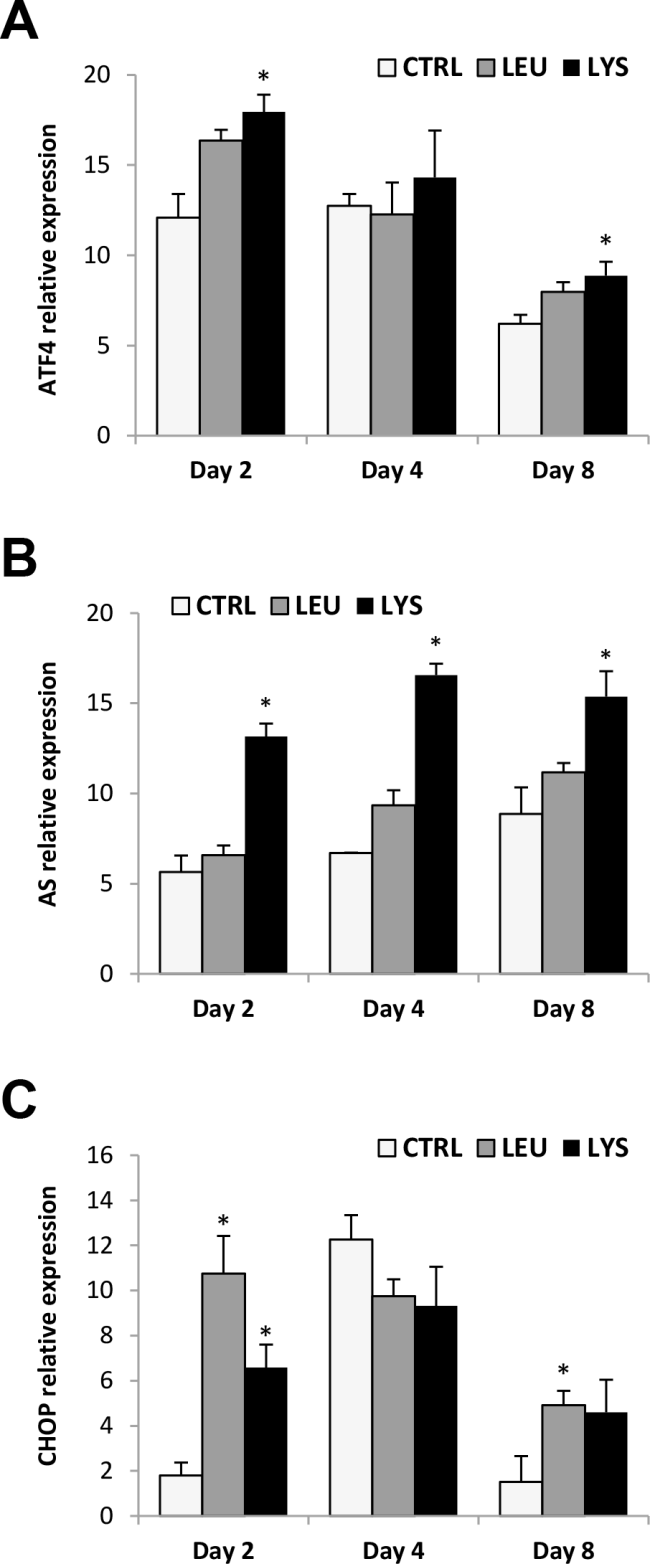
Effects of Lysine or Leucine deficient media on several AA limitation markers gene expression in gilthead sea bream cultured muscle cells. Quantitative relative expression of **(A)** ATF4, **(B)** AS, **(C)** CHOP normalized to the geometric mean of EF1α and RPS18 in myocytes at days 2, 4 or 8 after incubation with a growth medium control or deficient in Lys or Leu. Data are shown as means ± SEM (n = 3–4). Asterisks indicate significant differences compared to the control at each time (p<0.05). Different letters indicate differences for each group throughout the culture (p<0.05).

First of all, the gene expression of several AA limitation markers was studied. The results showed that the mRNA levels of the activating transcription factor 4 (ATF4) were increased at days 2 and 8 of culture with Lysine deficiency, but not at day 4 (Fig 1A). Regarding asparagine synthetase (AS), data showed a significant increase in gene expression at days 2, 4 and 8 in Lysine deficient medium, while the increase observed with Leucine deficiency was not significant (Fig 1B). Moreover, the CCAAT/enhancer-binding homology protein (CHOP) gene expression also increased at day 2 in response to both Lysine and Leucine deficiencies and at day 8 only in the case of Leucine (Fig 1C).

### 3.1. IGF system

**Fig 2:**
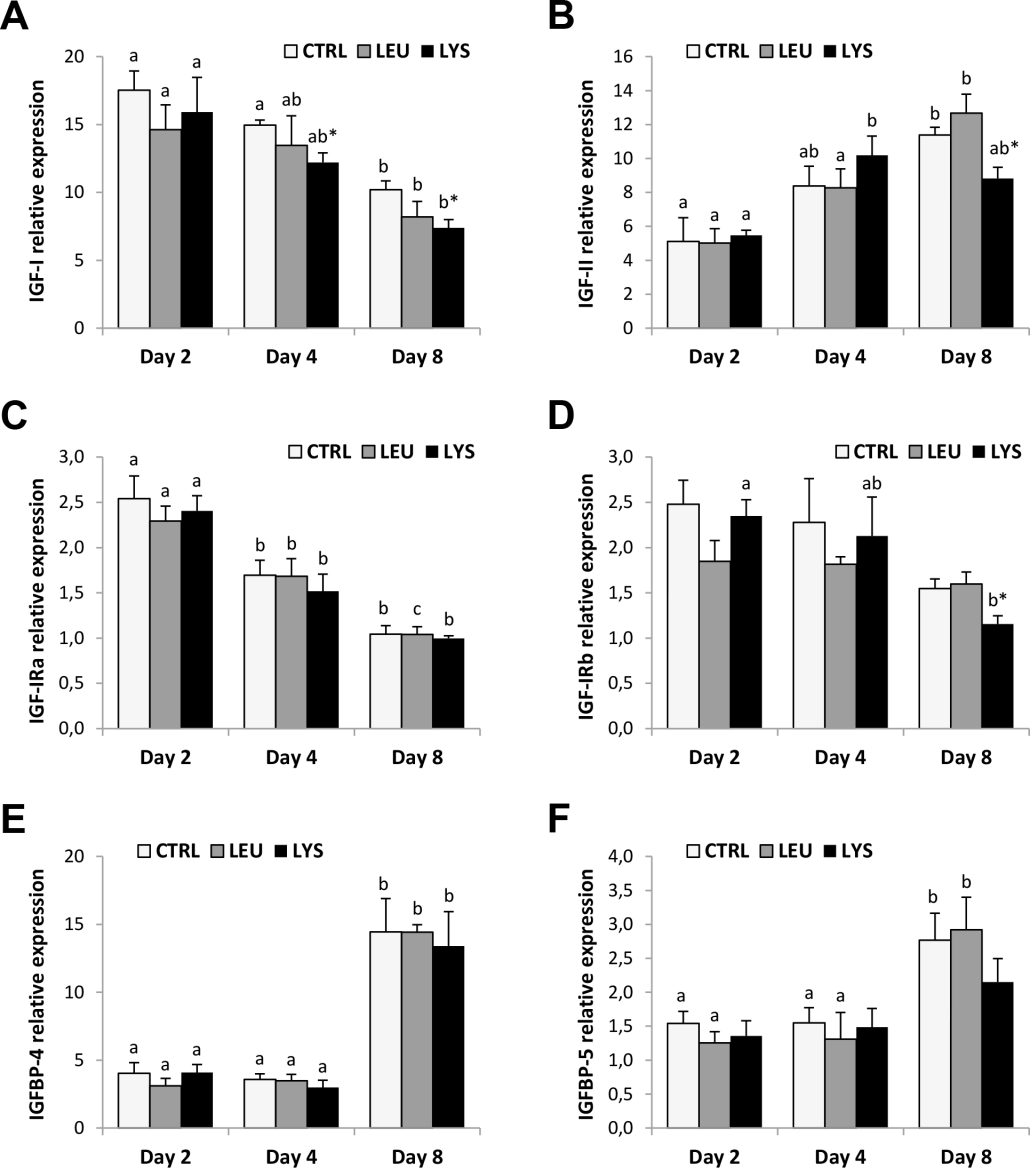
Effects of Lysine or Leucine deficient media on the IGF system genes expression in gilthead sea bream cultured muscle cells. Quantitative relative expression of **(A)** IGF-I, **(B)** IGF-II, **(C)** IGF-IRa, **(D)** IGF-IRb, **(E)** IGFBP-4 and **(F)** IGFBP-5 normalized to the geometric mean of EF1α and RPS18 in myocytes at days 2, 4 or 8 after incubation with a growth medium control or deficient in Lys or Leu. Data are shown as means ± SEM (n = 3–4). Asterisks indicate significant differences compared to the control at each time (p<0.05). Different letters indicate differences for each group throughout the culture (p<0.05).

IGF-I and IGF-II gene expression profiles through myocyte development were inverse, decreasing IGF-I expression and increasing IGF-II expression significantly in control conditions (Fig 2A and 2B). Similar patterns were observed in AA deficient media although IGF-I mRNA levels were significantly diminished with respect to the control condition at days 4 and 8 with the Lysine deficient medium. IGF-II expression did not reflect the deficiencies with the exception of Lysine at day 8 of culture that showed a significant lower transcript level compared to the control.

Regarding IGF-I receptors, both isoforms presented a decreasing profile in their gene expression during myocytes culture, although it was significant in all conditions for IGF-IRa but only significant in the cells cultured with Lys deficient medium in the case of IGF-IRb (Fig 2C and 2D). However, no effects were observed as a consequence of AA deficiencies, with the exception of Lysine that provoked a significant decrease of IGF-IRb at day 8.

Finally, IGFBP-4 and IGFBP-5 mRNA levels showed a parallel profile with significant increases at day 8 of culture compared to days 2 and 4 in all cases with the exception of the cells in Lysine deficient medium (Fig 2E and 2F).

### 3.2. MRFs and other markers (PCNA and MHC)

**Fig 3:**
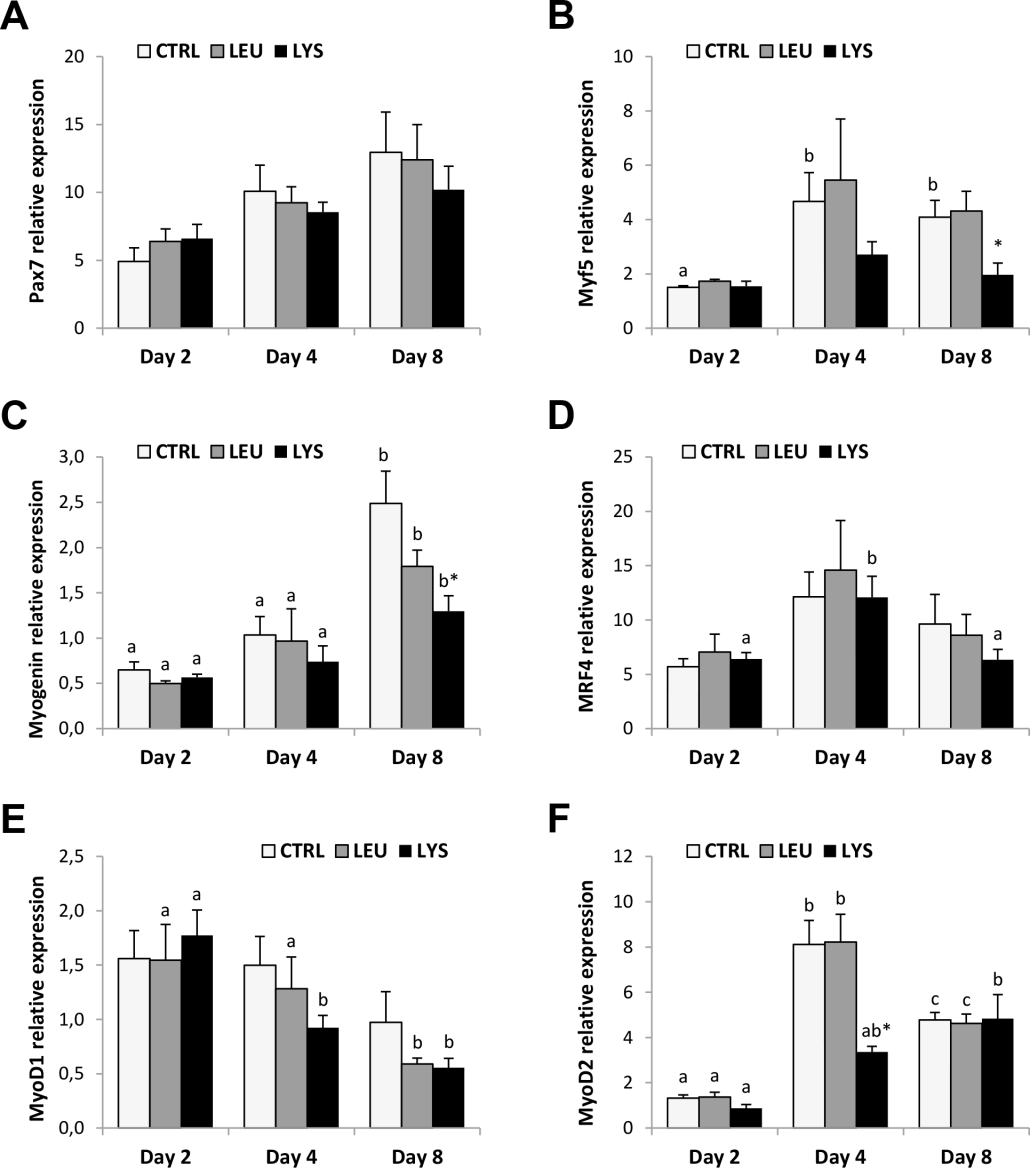
Effects of Lysine or Leucine deficient media on the MRFs genes expression in gilthead sea bream cultured muscle cells. Quantitative relative expression of **(A)** Pax7, **(B)** Myf5, **(C)** Myogenin, **(D)** MRF4, **(E)** MyoD1 and **(F)** MyoD2 normalized to the geometric mean of EF1α and RPS18 in myocytes at days 2, 4 or 8 after incubation with a growth medium control or deficient in Lys or Leu. Data are shown as means ± SEM (n = 3–4). Asterisks indicate significant differences compared to the control at each time (p<0.05). Different letters indicate differences for each group throughout the culture (p<0.05).

In the control condition, gene expression of Pax 7 did not present differences through culture development; while Myogenin expression was significantly higher at day 8 compared to days 2 and 4 in all conditions (Fig 3A and 3C). Myf5, MRF4 and MyoD2 showed highest mRNA levels at day 4 and then remained stable or decreased at day 8 showing significant differences only in some conditions (Fig 3B, 3D and 3F). MyoD1 significantly decreased trough the culture with the AA deficient media although it remained stable in the control condition (Fig 3E). Overall, AA deficiencies provoked in general a decrease of the MRFs gene expression compared to the control, more evident at day 4 and day 8 and, with stronger significant effects in Lysine deficient medium for Myf5, Myogenin and MyoD2.

**Fig 4:**
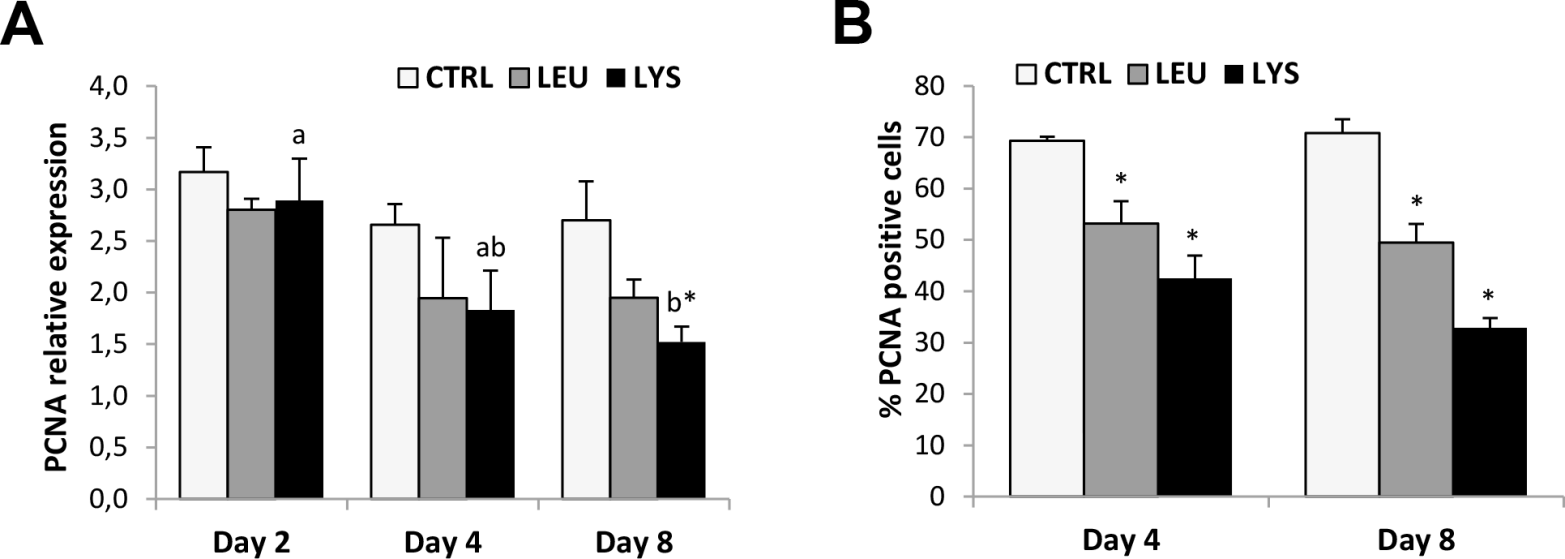
Effects of Lysine or Leucine deficient media on PCNA gene and protein expression in gilthead sea bream cultured muscle cells. Quantitative relative expression of **(A)** PCNA normalized to the geometric mean of EF1α and RPS18, and **(B)** quantification of PCNA-positive cells in myocytes at days 2, 4 or 8 after incubation with a growth medium control or deficient in Lys or Leu. Data are shown as means ± SEM (n = 3–4). Asterisks indicate significant differences compared to the control at each time (p<0.05). Different letters indicate differences for each group throughout the culture (p<0.05).

**Fig 5:**
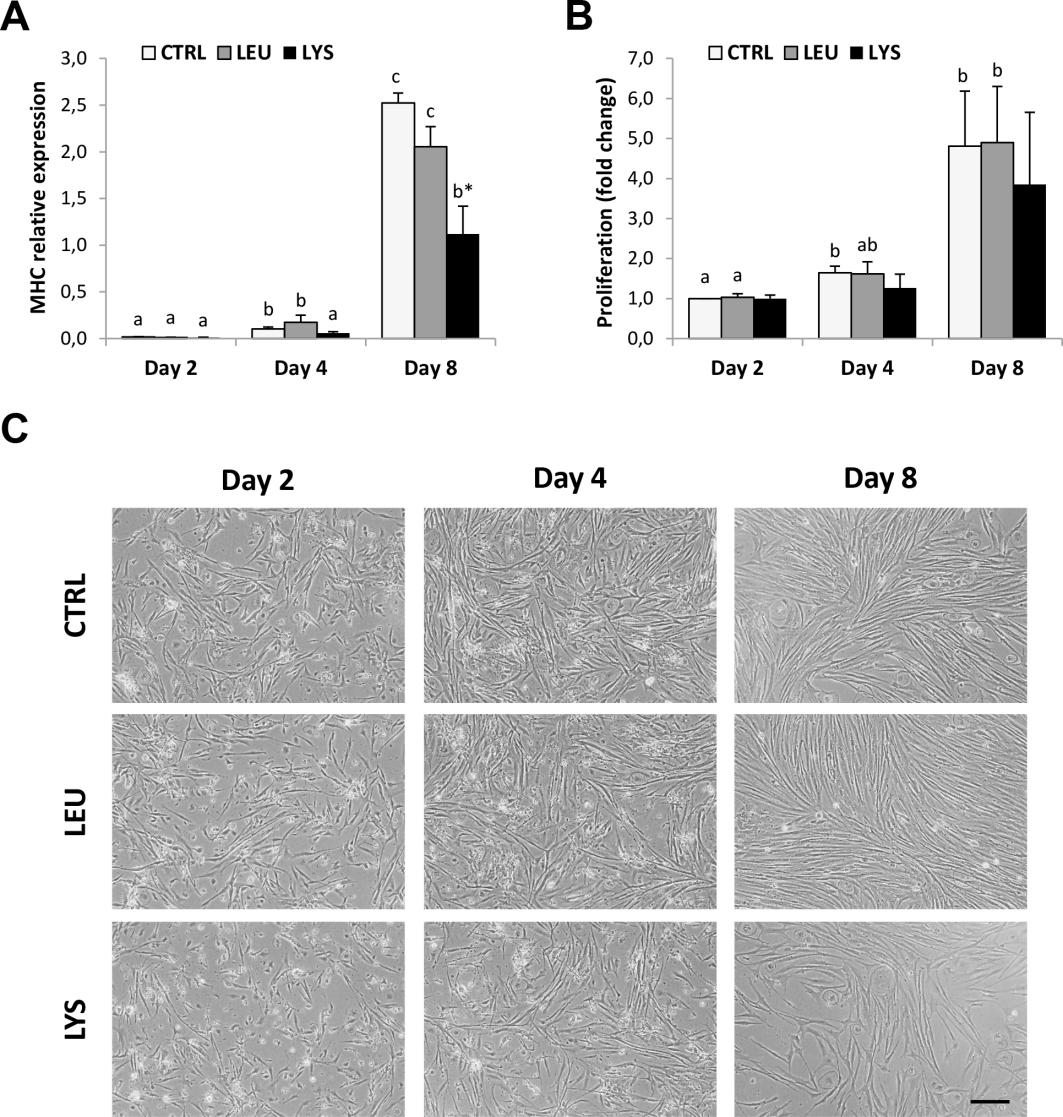
Effects of Lysine or Leucine deficient media on proliferation and differentiation markers expression in gilthead sea bream cultured muscle cells. Quantitative relative expression of (**A**) MHC normalized to the geometric mean of EF1α and RPS18, and proliferation measured by means of **(B)** MTT assay in myocytes at days 2, 4 and 8 after incubation with a growth medium control or deficient in Lys or Leu. Data are shown as means ± SEM (n = 3–4). Asterisks indicate significant differences compared to the control at each time (p<0.05). Different letters indicate differences for each group throughout the culture (p<0.05). **(C)** Representative images of gilthead sea bream cultured myocytes at days 2, 4 and 8 after incubation with a growth medium control or deficient in Lys or Leu. Objective: 10x. Scale bar: 50 μm.

The proliferation marker (PCNA) gene expression decreased during culture development with significantly lower values at day 8 compared to day 2 in the medium deficient in Lysine, which resulted in significant differences when compared to the control condition (Fig 4A). The same results were obtained for PCNA protein expression analyzed by quantifying the percentage of PCNA-positive cells by immunocytochemistry. In this case, both Lysine and Leucine deficient media showed significantly reduced levels compared to the control condition at both day 4 and day 8 (Fig 4B). Moreover, the gene expression of the differentiation marker, myosin heavy chain (MHC) increased trough the culture significantly with maximum levels at day 8 in all conditions; however, Lysine deficient medium showed significantly lower levels of expression than the control (Fig 5A). Furthermore, when looking at culture development by means of MTT assay, that increased significantly during culture in control and Leucine deficient conditions but not with Lysine deficiency supporting the previous data (Fig 5B).

Representative images of the cultures also showed evidence of a significantly reduced number of myocytes present in the Lysine deficient medium, whereas cells without Leucine appeared very similar to control cells (Fig 5C).

### 3.3. Signaling pathways

**Fig 6:**
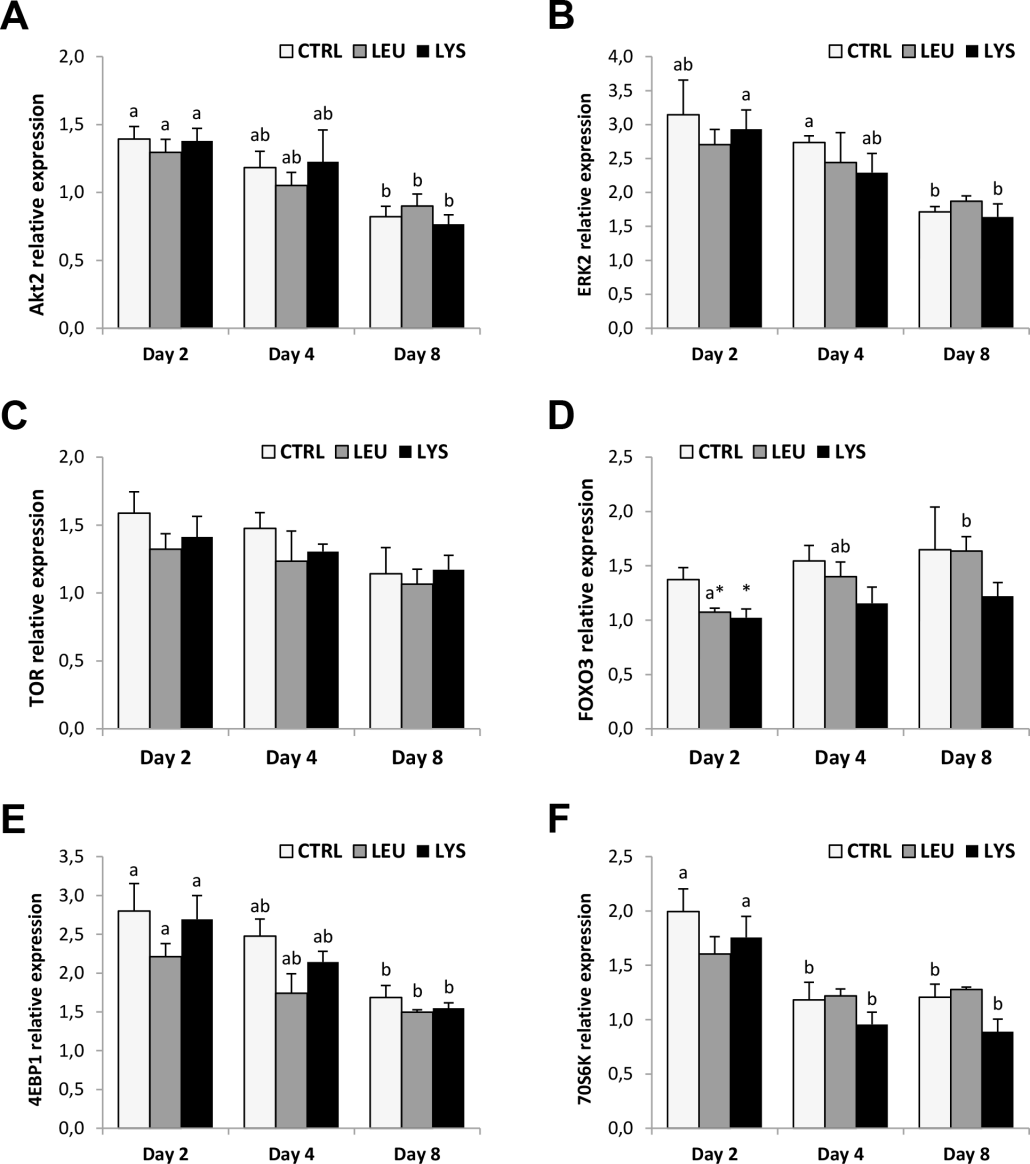
Effects of Lysine or Leucine deficient media on the signaling pathways genes expression in gilthead sea bream cultured muscle cells. Quantitative relative expression of **(A)** AKT2, **(B)** ERK2, **(C)** TOR, **(D)** FOXO3, **(E)** 4EBP1 and **(F)** 70S6K normalized to the geometric mean of EF1α and RPS18 in myocytes at days 2, 4 or 8 after incubation with a growth medium control or deficient in Lys or Leu. Data are shown as means ± SEM (n = 3–4). Asterisks indicate significant differences compared to the control at each time (p<0.05). Different letters indicate differences for each group throughout the culture (p<0.05).

In control conditions, the majority of the signaling molecules analyzed in this study, AKT2, ERK2, 4EBP1 and 70S6K, decreased their gene expression through the myocytes culture and significant lowest levels of mRNA were observed at day 8 (Fig 6A, 6B, 6E and 6F). On the other hand, TOR gene expression was constant, while FOXO3 showed a significant increase with Leucine limitation medium at day 8 (Fig 6C and 6D). AA deficiencies resulted in general in a decrease of gene expression parallel to that observed in control groups, highlighting the significant decrease observed at day 2 both in Lysine and Leucine deficient media with regards to FOXO3 when compared to the control.

## Discussion

The analysis of Lysine and Leucine final concentration in the media used in the present experiments have demonstrated that the deficiencies of both AA are comparable and remarkable respect to the control condition (i.e. 93.8%). Furthermore, the gene expression data of several AA limitation markers [33, 50–53] have indicated that the experimental conditions used resulted in important AA deficiencies affecting the status of the cells, although the effect of Lysine appeared to be more pronounced. Overall, this information permits to validate the experimental model used of Lysine and Leucine deficient media in order to further explore their effects in the development of gilthead sea bream muscle cells in culture.

### 4.1. IGF system during culture development and effects of AA deficiencies

In control conditions IGFs presented an inverse profile, with IGF-I showing a decrease and IGF-II an increase in gene expression through culture development. Jiménez-Amilburu et al. [46] described a similar profile of IGFs expression in the same species in a more detailed study, where IGF-I maintained high levels up to day 4 of culture to then decrease until day 12. IGF-II on the other hand, showed a second peak at day 10 of culture, suggesting that both growth factors may play different and complementary functions during myogenesis, as previously proposed [54].

The expression profile of IGF-I receptors and binding proteins in cultured growing myocytes in gilthead sea bream have not been previously reported. IGF-I receptors showed a gene expression profile similar to that of IGF-I, decreasing during culture, mainly for IGF-I-Ra. A similar pattern was found for IGF-IR1b in Atlantic salmon myocytes development, although the IGF-IR1a and IGF-IR2 isoforms showed a clear increase in their expression [36]. Receptor binding studies provide complementary information and Castillo et al. [55] and Monserrat et al. [27] found an increase in IGF-I binding during myocytes culture in rainbow trout and gilthead sea bream respectively, while Rosenthal et al. [56] reported a decrease in IGF-I binding when myoblast cells developed into myotubes on the mouse BC3H-1 muscle cell line. Nevertheless, binding reflects the interaction of the ligand with all receptor isoforms, which can perhaps respond in different directions. In fact, treatment of gilthead sea bream muscle cells with IGF-I provoked down-regulation in both isoforms expression, while IGF-II up-regulated IGF-IRb [45]. Differential responses between IGF-IR isoforms expression have been also reported by Chauvigné et al. [57] and Montserrat et al. [58] in fasting and refeeding conditions or by Gabillard et al. [59] by temperature treatments in rainbow trout. Such variance could thus suggest different roles for each isoform to allow amplifying the regulatory capacity of the fish muscle depending on the physiological situation.

Regarding IGFBPs, in our study mRNA profiles of IGFBP-4 and IGFBP-5 were similar, increasing significantly through culture development except in the case of Lysine deficiency for IGFBP-5. In agreement with our data, in murine C2 myoblasts it is well-known that IGFBP-5 transcription is highly activated during myoblast differentiation [60, 61]. Moreover, Bower and Johnston [36] found that IGFBP-4 showed a significant increase during Atlantic salmon myocytes growth with a peak at day 8 of culture, as that found in our model. However, in their study both IGFBP-5 isoforms showed maximal levels at day 2 to then decrease to very low levels at day 8 that were maintained up to day 20. In a previous study, Azizi et al. [45] found in gilthead sea bream myocytes that IGFBP-5 expression (but not IGFBP-4) increased when cells were treated with IGF-II. This response could explain that in the present study the maximum level of IGFBP-5 coincides with the peak of IGF-II at day 8. This agrees with the recognized stimulatory function and cross-regulation of IGFBP-5 with IGF-II during myogenesis as previously described [24, 62].

Respect to the impacts of AA deficiencies on IGF system members gene expression, the main effect observed was the decrease of IGF-I expression at days 4 and 8 and that of IGF-II at day 8 with Lysine deficiency. IGF-IRb gene expression also decreased significantly at day 8 in the absence of Lysine. Overall this indicates that Lysine deficiency seems to be compromising the function of IGFs in these muscle cells.

In fish, there is very little information about the effects of AA deficiencies over the IGF system. As far as we know, this is the first study that checks the effects of Lysine or Leucine deficiencies on fish muscle cells cultured *in vitro*. Most of the studies up to date were based on the effects of AA supplementation or deficiency in fish diets *in vivo*, where several studies have shown the importance of keeping a required level depending on the species of the different essential AA in the aquafeeds for adequate growth and health [7, 10, 13, 63, 64]. Very recently, Rolland et al. [12] have shown that the transcript levels of IGF-I but not IGF-II in liver increased linearly with the rise of dietary Methionine in rainbow trout and a similar result was observed in Atlantic salmon with increasing dietary Lysine [65]. *In vitro*, Lansard et al. [40] analyzed the effects of Leucine, Methionine and Lysine stimulation in the regulation of intermediary metabolism-related genes expression in rainbow trout hepatocytes and reported that Leucine had effects similar to a pool of AA while Lysine only had limited effects. Regarding muscle *in vivo*, Hevrøy et al. [65] observed that high Lysine intake resulted in a 7-fold up-regulation of muscle IGF-II mRNA levels in Atlantic salmon. Contrary, low Lysine intake decreased the nitrogen deposition and muscle protein accretion and significantly down-regulated muscle IGF-II, as well as IGF-I expression is reduced in fasted fish [58, 66]. IGFs provide a mean of controlling cell proliferation and differentiation and have a biological effect on muscle growth [27, 39, 46, 54]. These findings together with our results fit in the notion that Lysine seems to be an important local anabolic regulator of muscle tissue development in gilthead sea bream.

Muscle IGFBP-4 and IGFBP-5 were not affected by Lysine or Leucine deficiencies in our study. Hevrøy et al. [65] found that liver IGFBP-1b was down-regulated in response to low Lysine intake, which may be linked to the catabolic status of the fish. On the other hand, after AA stimulation, Bower and Johnston [36] reported increased expression of both IGFBP-5 isoforms indicating the promotion of an anabolic situation. In the study of Hevrøy et al. [65], mRNA levels of muscle IGF-IR were not affected by Lysine intake, while in our experiment only the gene expression of IGF-IRb in day 8 cells was decreased by the deficiency of the same AA, suggesting differential effects for both IGF-IRs in response to Lysine limitation in myocytes. All this points out that the deficiency of a single AA, either Lysine or Leucine, is not enough to determine significant changes on the gene expression of IGFBPs and IGF-IRa in these cells and that a more dramatic limitation such as complete suppression of AA like during fasting might be required.

### 4.2. Effects of AA deficiencies in MRFs, PCNA and MHC

The gene expression profile observed in control conditions for MRFs and other myogenic markers is basically in agreement with previous studies in *in vitro* muscle cells development in the same species by our group [66] or other species like Atlantic salmon [67]. In fact, MyoD1 and PCNA showed highest gene expression at the beginning of myocytes development, while Myogenin increased with the progression of the culture in parallel with the structural component, also used as a marker of myocytes differentiation, MHC. The little differences observed on some profiles, like Myf5 and MRF4, can be consequence of the variability of cultures, the slightly different media used at some points or the characteristic physiological stage of the fish utilized to obtain the satellite cells among the different studies.

AA deficiencies provoked a clear effect on myocytes development, especially in the case of Lysine. A clear down-regulation in gene expression for MyoD2 at day 4 and Myf5, Myogenin, PCNA and MHC at day 8 in the absence of Lysine is observed, and although lack of Leucine did not show significant differences, it presented similar trends. Lysine and Leucine deficiencies appear to retard cell proliferation and muscle differentiation, as supported by the observed significant decrease in PCNA protein expression. In fact, in a recent study by our group [39], AA treatment alone or in combination with IGF-I determined an increase in PCNA and Myogenin gene expression in gilthead sea bream cultured myocytes in agreement with these results. Moreover, although the results were not significantly different, the proliferation assay (MTT) indicated decreased proliferation also in the condition without Lysine. In this sense, also the different representative images of the status of the culture clearly show reduced number of cells in the condition without Lysine. Moreover, Lysine deficiency also clearly affected myocyte differentiation, as indicated by the minor increase in Myogenin gene expression during development. Myogenin has been described to show a peak on gene expression at day 8 of culture coinciding with the process of myocyte differentiation and fusion into myotubes [66]. Therefore, the significant differences observed at this point between the control and the Lysine deficient medium make sense as this would be the most critical time with regards to Myogenin activation and corroborate an important role for this AA also in differentiation. On the other hand, the fact that Leucine did not affect the expression of any gene analyzed as well as did not show clear evidence of reduced myogenesis in the photographs of the cultures, indicates that the importance and mechanism of action of both AA is different. Thus, Leucine may have minor effects acting at a post-transcriptional level to regulate myocytes proliferation, whereas Lysine that affected the transcription factors gene expression could be also at the same time altering other important myogenic genes expression; therefore, provoking a stronger effect on muscle growth.

Bower and Johnston [67] observed in fasted salmon myocytes, lower levels of MyoD paralogs in comparison to AA-treated cells. Averous et al. [33] reported also an up-regulation of Myf5 mRNA and protein level and a decrease of MyoD protein level but not mRNA during Leucine starvation in C2C12 myoblasts. A similar effect on MyoD1 and MyoD2 was observed in our study with a gene expression decrease in AA deficient media. In that same study with murine cells, MHC protein expression was induced during differentiation in the presence of Leucine, whereas during Leucine starvation MHC protein expression was absent. However in our study, it is noticeable that Lysine affected more importantly than Leucine to myocytes development with diminution on the expression of Myf5, Myogenin, PCNA and MHC genes expression. This could be a particular response of gilthead sea bream to Lysine deficiency and stimulates future research with regards to the requirements of this particular AA in the diets of this species.

All that points out the important effects of AA in myocytes function and it agrees with the *in vivo* study in rainbow trout by Alami-Durante et al. [68], which demonstrates that changes in dietary essential AA have significant effects in fish myogenesis.

### 4.3. Signaling pathways molecules during culture development and effects of AA deficiencies

Similar gene expression profiles were found in the present study for both AKT2 and ERK2, decreasing through the culture very closely to that observed for IGF-I, which makes sense considering that IGF-I transduces its signal mainly through these two pathways. TOR gene expression was stable but its downstream effectors, 4EPB1 and 70S6K, showed significantly lower gene expression either at day 4 or at day 8 compared to day 2. There is no information in fish muscle cells regarding these profiles but in mammals is well characterized the significant role of TOR at the beginning of the myogenesis [69]. Finally, FOXO3 in control conditions was stable through the culture. We have not found FOXO3 data describing its profile in cultured fish muscle cells, but in mammalian C2C12 cells (at the protein level), the increase of FOXO isoforms phosphorylation in the stage of myotubes respect to myocytes has been well described [70]. Moreover, these data are concordant with the decrease of AKT2 during culture, a well-known inhibitor of FOXO3 activation.

Regarding the treatments, AKT2, ERK2 and TOR genes expression was not affected by AA deficiencies in this study. Vélez et al. [39] observed in the same model that a treatment with a cocktail of AA increased TOR gene expression and phosphorylation and similar results were obtained in Atlantic salmon myoblasts [71]. Certainly, it is well recognized the key role of TOR regulating protein synthesis according to AA levels [34]. Leucine stimulates protein synthesis in muscle by up-regulating TOR signaling and S6K1 phosphorylation [72]. Averous et al. [33] found also in C2C12 myoblasts that Leucine starvation mimicked the effects observed with rapamycin treatment on TOR, showing a decrease in phosphorylation in the downstream molecules of this signaling system. In a different study with diabetic-induced rats, Leucine did not affect TOR signaling through 4EBP1 or 706SK phosphorylation although protein synthesis was stimulated [73]. The authors suggested that Leucine also participates in the activation of protein synthesis via an insulin-independent mechanism that remains unknown. In rainbow trout hepatocytes, Lansard et al. [40] showed that single AA do not have the capacity to increase neither AKT nor TOR phosphorylation. In this sense, the absence of a response of TOR, 4EBP1 and 70S6K genes expression detected in gilthead sea bream could be related to the well-known resistance of fish to starvation compared to mammals. Although we could also consider that the effects on AKT2, ERK2 and TOR gene expression require more dramatic or prolonged deficiencies than those used in the present study; regulation at the level of protein phosphorylation cannot be excluded. As observed, it seems that the IGF system members or the MRFs gene expression respond to the treatment; therefore, a different time might be necessary to detect changes in the gene expression of signaling pathways molecules in response to AA deficiencies in fish cells or perhaps these changes might be occurring at a post-transcriptional level and, it would be very interesting to verify whether changes in AKT and TOR phosphorylation occur as a consequence of a single AA deficiency in gilthead sea bream.

FOXO3 gene expression responded to Lysine and Leucine deficiencies in gilthead sea bream myocytes showing a decrease, mainly at day 2. It is well-known in mammals that FOXO1 and FOXO3 are downstream targets of the IGF-AKT pathway. They can enhance autophagy-related genes in muscle, being sufficient to activate this process causing muscle degradation [74, 75]. In a catabolic situation like nutrient starvation, we would expect an increase in FOXO3 phosphorylation and therefore an increase in the autophagy flux. In the last years Seiliez et al. [35, 76] have demonstrated that starvation enhances the expression of autophagy-related genes in both *in vivo* muscle and *in vitro* cultured myocytes of rainbow trout. In these works, the authors have also shown that FOXO system is not involved in the regulation of autophagy activation in muscle of rainbow trout as an effect of AA availability. Altogether these and our results suggest a differential role for FOXO in fish muscle that has not been completely elucidated.

## Conclusions

In summary, we can conclude that AA deficiencies affect several important components of IGF system and myogenesis regulators and especially Lysine seems to present a significant role in gilthead sea bream muscle growth. Consequently, new diets formulation should take into account the requirement of this AA in this species. At the same time, this model brings new insights into the role of IGFs, MRFs, FOXO3 and TOR pathways regulating fish myocytes growth.
